# Supplementary material for: “If You Do Not Take the Medicine and Complete the Dose…It Could Cause You More Trouble”: Bringing Awareness, Local Knowledge and Experience into Antimicrobial Stewardship in Tanzania
Source: Antibiotics (Basel). 2023 Jan 25;12(2):243. doi: 10.3390/antibiotics12020243 (PMC9952761; doi:10.3390/antibiotics12020243)
Supplement: Supplementary file 1 [file antibiotics-12-00243-s001.zip › antibiotics-1991464-supplementary.pdf]

SNAP-AMR

Version 1: Community Animal Health Workers/Local Livestock Health Experts In-depth Interview Schedule

GENERAL INFORMATION/TAARIFA ZA JUMLA

Name of interviewer **Jina la muongoza mahojiano:** ______________________________
Date **Tarehe:** ____
Name of Interviewee **Jina la mshiriki:** _______________________________
Name of Interviewee's Institution **Jina la taasisi ya mshiriki:** ___________
Highest Level of Education **Kiwango cha juu cha elimu:** ________________
Age **Umri:**____
Gender **Jinsia:** ____
Name of District **Jina la Wilaya:** _______________________________
Name of Ward **Jina la Kata:** __________________________________
Name of Village **Jina la Kijiji:** _________________________________
Name of Sub-village **Jina la Kitongoji:** __________________________
Position of Participant Giving Information **Nafasi ya mshiriki anayetoa taarifa:**

1. Community Animal Health Worker **Mtoa huduma ya afya ya jamii kwa mifugo[ ]**
2. Local Livestock Expert **Mtaalamu wa mifugo katika eneo** [ ]
3. Other (please specify) **Nyingine (tafadhali bainisha)** [ ] ____________________

1. PERSONAL MOTIVATION/MOTISHA BINAFSI

1.1 How long have you worked/engaged in the livestock health sector, and how long in this ward/district? **Ni kwa muda gani umefanya kazi/kujihusisha katika sekta ya afya ya mifugo, na ni kwa muda gani katika Kata/Wilaya hii?**

1.2 Why did you choose to work/engage in the livestock health sector? **Ni kwanini ulichagua kufanya kazi/kujihusisha katika sekta ya afya ya mifugo?**

1.3 What do you enjoy most about working/being engaged in the livestock health issues? **Ni kitu gani unafurahia zaidi kuhusu kufanya kazi/kuhusishwa katika masuala ya afya ya mifugo?**

1.4 Is this the only work that you do? If no, what other income generating activities do you engage in? (Probe if they engage in other livestock related businesses e.g. veterinary shops, livestock trade) **Je, hii ni kazi pekee ambayo unaifanya? Kama hapana, unajishughulisha na nini kingine kwa ajili ya kuongeza kipato chako? (Dadisi kama wanajihusisha na biashara nyingine zinazohusiana na mifugo mfano duka la dawa za mifugo, biashara ya mifugo)**

1.5 What training have you had for community animal health work? **Je, Una mafunzo gani juu ya uhudumu wa afya ya mifugo katika jamii?**

1.6 What are the biggest challenges you have faced when working/engaging on livestock health? **Ni changamoto zipi kubwa umekutana nazo wakati wa kufanya kazi/ kujihusisha kwenye afya ya mifugo?**

1.7 How do you think these challenges can be resolved? **Ni namna gani unafikiri hizo changamoto zinaweza kutatuliwa?**

1.8 What are your expectations for the Tanzanian veterinary health system in the next decade, in terms of the hopes you have for it? **Ni yapi matarajio/matumaini ulionayo kwa mfumo wa afya ya mifugo Tanzania katika miaka kumi ijayo?**

1.9 What are the expectations you have for the Tanzanian veterinary health system in the next decade, in terms of the fears you have for it**? Ni hofu gani ulionayo kwa mfumo wa afya ya mifugo Tanzania katika miaka kumi ijayo?**

1.10 How efficient do you think that the veterinary health system in meeting the needs of the farmers? Why? **Ni kwa namna gani unafikri mfumo wa afya ya mifugo una ufanisi katika kuyafikia mahitaji ya wafugaji? Kwanini?**

1.11 What do you think are the farmers' opinions about the livestock health services provided to them? **Ni nini maoni ya wafugaji juu ya huduma za afya ya mifugo zinazotolewa kwao?**

General questions

3.1 How do you determine the dosage of anti-biotics/medicine to recommend/prescribe for livestock? **Ni kwa namna gani unaamua dozi ya antibiotiki/ madawa kwa ajili kupendekeza / kuandika kwa ajili ya mifugo?**

3.2 Do community members usually adhere to the treatment prescribed/recommended for their livestock in terms of dosage and days of administration? (If yes, skip to 3.20) **Je, wanajamii wanafuata matibabu wanayoandikiwa/ kupendekezwa kwa mifugo yao kwa kuzingatia dozi na siku za matumizi? Kama ndiyo enda 3.20)**

3.3 If no, how would you rate the problem of non-compliance? *(Out of 10 persons, how many will not comply to treatment requirements?)* **Kama hapana, je unatathimini vipi kiwango cha kutofuata? *(Kati ya watu kumi, wangapi hawatafauta maagizo ya tiba)***

3.4 What are the reasons why non-adherence to medicines may be happening in the community? **Ni sababu gani inapelekea kutofuata maelekezo ya dawa katika jamii?**

3.5 Are there any dangers in non-adherence of medicines? If yes, can you explain what they are? **Je, kuna hatari zozote za kutofuata maelekezo ya dawa? Kama ndiyo, unaweza kuelezea ni zipi?**

3.6 Are there instances in which farmers insist on prescription/recommendation of particular medicines for livestock? Which medicines are these? **Je, kuna wakati**

wafugaji wanasisitiza kuandikiwa/kupendekeza dawa fulani kwa ajili ya mifugo? Ni dawa zipi hizo?

3.7 As a livestock health service provider, what do you do in such cases? **Kama mtoa huduma ya afya ya mifugo, unafanya nini kwa matukio haya?**

3.8 How would the farmers respond if you did not prescribe for them the medicine they wanted? **Wafugaji wanapokeaje endapo mtoa huduma hajawaandikia dawa wanayoitaka?**

3.9 Do you think antibiotics are overprescribed/over recommended by livestock health providers? If yes/no why? **Je, unadhani antibiotiki zinaandikwa/ kupendekezwa zaidi na watoa huduma wa afya kwa mifugo? Kama ndio/hapana kwanini?**

3.10 Do you think antibiotics are overused in the community/by farmers? If yes/no why? **Je, unadhani antibiotiki zinatumika sana katika jamii/ na wafugaji? Kama ndiyo/hapana kwanini?**

3.11 How do you dispose medicines that are expired? **Ni kwa namna gani wanaondoa/teketeza dawa zilizopitwa na wakati?**

3.12 Do you think the medicines given to livestock have any effects on the environment? If yes, in what ways? **Je, madawa yanayopewa mifugo yana madhara yoyote kwa mazingira? Kama ndio, kwa njia gani?**

3.13 As a farmer, do you feel you always have access to all the suitable antibiotics/medicines you would like to use or are there times when certain antibiotics are unavailable? (If yes skip to 3.29). **Kama mfugaji, je ni wakati wote kunakuwa na upatikanaji unaofaa wa antibiotiki/dawa mnazopendelea kutumia? (kama ndio nenda 3.29)**

3.13.1 Probe If drugs are not available: What do you do if they do not have the required antibiotics in stock? ***Dadisi kama madawa hamna: unafanyaje kama antibiotiki wanazohitaji hazipatikani kwenye akiba?***

3.13.2 *Probe if drugs are not available: How do you feel if stock is unavailable?* **Dadisi kama dawa hazipo: unahisi vipi kama akiba haipo?**

3.29 Have you heard of the term "bacteria"? If so, can you explain to us what it is? Provide examples of diseases caused by bacteria? **Je umeshawahi kusikia neno "bakteria"? kama ndiyo unaweza kutuelezea ni nini? Toa mifano ya magonjwa yanayo sababishwa na bacteria.**

3.29.1 Do you know what kinds of medicines are used to treat bacteria? **Je unajua aina ya madawa yanayotumika kutibu 'bakteria'?**

3.30 Have you heard of the term "virus"? If so, can you explain to us what it is? Provide examples of diseases caused by viruses? **Je umeshawahi kusikia neno "kirusi"?**

kama ndiyo unaweza kutuelezea ni nini? Toa mifano ya magonjwa yanayosababishwa na kirusi?

3.30.1 Do you know what kinds of medicines are used to treat viral infection? **Je unajua aina gani ya dawa zinazotumika kutibu maambukizi ya kirusi?**

4: AMR KNOWLEDGE UELEWA WA USUGU WA VIMELEA

4.1 Are there any medicines that you prescribe/recommend or farmer's use that are no longer effective even after use within the required guidelines? If yes, which ones are they? (probe for name/brand/manufacturers) (If no skip to 4.4) **Je, kuna dawa ambazo umeandika/kupendekeza au wafugaji wanatumia na sasa zimerepotiwa hazifanyi kazi vizuri hata baada ya kutumia miongozo inayohitajika? Kama ndiyo, ni zipi? (dadisi kujua jina/ aina/ mtengenezaji) (Kama la, nenda 4.4)**

4.2 For what condition(s)/disease(s)/uses are they not effective?

Ni kwa hali gani/magonjwa/matumizi ambayo yametolewa taarifa kuwa hazifanyi kazi vizuri?

4.3 Why are they considered no longer effective? **Ni kwanini inachukuliwa kuwa hazifanyi kazi vizuri tena?**

4.4 How are you resolving/dealing with the challenge of the ineffective medicines at community level? **Ni namna gani unatatua/ utatatua changamoto ya madawa kutoweza kufanya kazi vizuri katika ngazi ya jamii?**

4.5 Have you heard the term "antibiotic"? Je umeshawahi kusikia neno "Antibiotiki"?

4.5.1 If so, what kinds of illnesses do antibiotics treat? **Kama ndiyo ni aina gani ya magonjwa antibiotiki inatibu?**

4.5.2 What are the most commonly used antibiotics in your community? **Ni antibiotiki gani kwa kawaida inatumika mara kwa mara katika jamii yako?**

4.6 *Ask only if they know what antibiotics are:* Have you ever heard of the term "antibiotic resistance", if so can you tell us what it means**? (if they know skip to 4.8) Uliza tu kama wanajua antibiotiki ni nini: Je umeshawahi kusikia neno "usugu wa antibiotiki"? kama ndiyo unaweza kutuambia nini maana ya usugu wa antibiotiki? (kama hapana nenda 4.8)**

4.7 If they do not know what antibiotics are: Are you aware of 'drug resistance'? If so, can you tell us what it means? **Kama hawaelewi antibiotiki ni nini: je unaelewa usugu wa madawa ni nini? Kama ndiyo unaweza kutuambia nini maana ya usugu wa madawa**?

4.8 Is it (anti-biotic/drug resistance) an issue that can affect livestock? If so, how? **Je, unadhani (usugu wa antibiotiki/madawa) ni jambo ambalo linaweza likaathiri mifugo? Kama ndiyo, ni kwa namna gani?**

4.9 What do you think are the main causes of anti-biotic/drug resistance in livestock? Je **unadhani ni nini kinasababisha usugu wa antibiotiki/madawa kwa mifugo?**

4.10 As a livestock health care practitioner, do you feel your individual actions can contribute to *spreading* antibiotic/drug resistance, and if so, in what ways? **Kama mtoa huduma wa afya kwa mifugo unadhani matendo yako binafsi yanaweza kuchangia kusambaa kwa usugu wa antibiotiki/madawa, kama ndiyo ni kwa namna gani?**

4.11 As a livestock health care practitioner, do you feel your individual behavior can contribute to *preventing* antibiotic/drug resistance, and if so, in what ways? **Kama mtoa huduma ya afya kwa mifugo, unafikiri tabia yako binafsi inaweza kuchangia katika kuzuia usugu wa antibiotiki, kama ndiyo, kwa namna gani?**

4.12 Do you ever consider antibiotic/drug resistance when you are prescribing/recommending antibiotics for livestock? **Je, huwa unazingatia usugu wa antibiotiki/madawa wakati unapoandika/pendekeza antibiotiki kwa mifugo?**

4.13 Do you think the people you serve understand antibiotic/drug resistance? **Je, unadhani watu unaowahudumia wanaelewa usugu wa antibiotiki/madawa?**

4.14 If you were to give advice the community on anti-biotic/drug resistance, what challenges are you likely to face? **Iwapo ungetakiwa kutoa ushauri kwa jamii kuhusu usugu wa antibiotiki/madawa, ni changamoto zipi unatarajia kukutana nazo?**

4.15 Have you received training and/or information regarding anti-biotic resistance? **Umeshapokea mafunzo na/au taarifa kuhusiana na usugu wa antibiotiki?**

> *4.15.1 Probe, if yes: Who provided the* ***training****, when was the training taken, what kind of information was given, what did you think of it, and did it change any of your practices?* ***Dadisi, kama ndiyo: Nani alitoa mafunzo, mafunzo yalifanyika lini, ni aina gani ya maelezo yalitolewa, unafikiri nini kuhusu hilo, na je ilibadili utendaji wako?***
>
> *4.15.2 Probe if no: Would you like to receive training or information, and if so what format would be most useful?* ***Dadisi, kama hapana: Utapendela kupokea mafunzo au taarifa, na kama ndiyo ni mfumo upi utakuwa wenye ufanisi?***

4.16 What do you think will happen if antibiotic resistance is not dealt with? **Ni nini matokeo yanayotarajiwa ya usugu wa antibiotiki kama halitoshughulikiwa?**

4.17 Who should be responsible for tackling anti-biotic/drug resistance in livestock? **Ni nani anawajibu wa kushughulikia usugu wa antibiotiki/madawa kwa mifugo?**

> *4.17.1 Probe: Is it an issue at the* ***community level or national level****? And why should it be their responsibility?* ***Dadisi: Je hili ni tatizo katika ngazi ya jamii au taifa? Na kwanini inapaswa kuwa wajibu wao?***

4.18 In what ways can antibiotic/drug resistance be curbed in the livestock sector? **Ni kwa njia zipi usugu wa antibiotiki/madawa unaweza kuzuiwa kwenye sekta ya mifugo?**

4.19 Are you aware of the Tanzania National Action Plan on AMR? **Je, unafahamu juu ya mpango mkakati wa Kitaifa juu ya usugu wa vimelea Tanzania?**

> 4.19.1 If yes, how did you come to hear about it.? **Kama ndiyo, ulifahamu vipi?**
>
> 4.19.2 Have you read it? **Je umeshaisoma?**

4.19.3 If they have read, what have you learnt from it? **Kama umeshasoma, umejifunza nini?**

4.19.4 Has your current practice changed in any way because of the NAP, if so in what way? **Je, utendaji wako wa sasa umebadilika kwa namna yoyote kwa sababu ya mpango mkakati wa Taifa? Kama ndiyo, kwa namna gani?**

Thank you for participating! Nashukuru kwa kushiriki
